# Biological age as a useful index to predict seventeen-year survival and mortality in Koreans

**DOI:** 10.1186/s12877-016-0407-y

**Published:** 2017-01-05

**Authors:** Jinho Yoo, Yangseok Kim, Eo Rin Cho, Sun Ha Jee

**Affiliations:** 1 Bioage Medical Research Institute, Bio-Age Inc., Seoul, Republic of Korea; 2 College of Oriental Medicine, Kyung Hee University, Seoul, Republic of Korea; 3 Department of Epidemiology and Health Promotion, Institute for Health promotion, Yonsei University Graduate School of Public Health, Seoul, Republic of Korea

**Keywords:** Biological age, Biomarkers, Chronological age, Cox regression, Mortality, Risk of death

## Abstract

**Background:**

Many studies have been conducted to quantitatively estimate biological age using measurable biomarkers. Biological age should function as a valid proxy for aging, which is closely related with future work ability, frailty, physical fitness, and/or mortality. A validation study using cohort data found biological age to be a superior index for disease-related mortality than chronological age. The purpose of this study is to demonstrate the validity of biological age as a useful index to predict a person’s risk of death in the future.

**Methods:**

The data consists of 13,106 cases of death from 557,940 Koreans at 20–93 years old, surveyed from 1994 to 2011. Biological ages were computed using 15 biomarkers measured in general health check-ups using an algorithm based on principal component analysis. The influence of biological age on future mortality was analyzed using Cox proportional hazards regression considering gender, chronological age, and event type.

**Results:**

In the living subjects, the average biological age was almost the same as the average chronological age. In the deceased, the biological age was larger than the chronological age: largest increment of biological age over chronological age was observed when their baseline chronological age was within 50–59 years. The death rate significantly increased as biological age became larger than chronological age (linear trend test, *p* value < 0.0001). The largest hazard ratio was observed in subjects whose baseline chronological age was within 50–59 years when the cause was death from non-cancerous diseases (HR = 1.30, 95% confidence intervals = 1.26 - 1.34). The survival probability, over the 17 year term of the study, was significantly decreased in the people whose biological age was larger than chronological age (log rank test, *p* value < 0.001).

**Conclusions:**

Biological age could be used to predict future risk of death, and its effect size varied according to gender, chronological age, and cause of death.

**Electronic supplementary material:**

The online version of this article (doi:10.1186/s12877-016-0407-y) contains supplementary material, which is available to authorized users.

## Background

Many studies have been conducted to quantitatively estimate biological age using measurable biomarkers since a study by Comfort in 1969 [1]. Biological age can represent a person’s aging status more appropriately compared to chronological age because, while chronological age is just a period of living which does not consider person’s health status, biological age is associated with health status, which is closely related with aging. There are no standardized or widely accepted sets of biomarkers for estimating biological age; nonetheless, several researchers have suggested some criteria that should be satisfied by biomarkers of aging [2–4], which included age-related change, nonlethal measurability, essential factor for health, fundamental reflection of biological or bodily process, and highly reproducibility. By considering these criteria, some clear set of requirements for biomarkers has been discussed in the research by Sharman and Zhumadilov [5]: 1) provide information about the functional condition of the body, its metabolic and regulatory systems, 2) have quantitative characteristics that correlate with age, 3) be reproducible, sensitive, and specific, 4) be suitable for use in humans as well as in laboratory animals. Since the biological age study began, by considering these criteria, various biomarkers including physical, physiological, or biochemical parameters have been used to compute biological age [6–10]. Some biomarkers were commonly found in these studies: body mass index (BMI), blood pressure (BP), waist circumference (WC), forced expiratory volume in 1 s (FEV1), body muscle percentage (BMP), body fat percentage (BFP), blood urea nitrogen (BUN), albumin, globulin, high density lipoprotein (HDL), low density lipoprotein (LDL), and/or triglyceride (TG), which are all relatively easily obtainable in a general health check-up. BMI, BP, and WC are associated with physical fitness, and physical strength could be represented by FEV1, BMP and/or BFP. Meanwhile, BUN, albumin, globulin, HDL, LDL, and TG are associated with biochemical factors.

Appropriate statistical algorithm is essential for computing biological age. Currently, two statistical algorithms have been introduced and widely used: multiple linear regression (MLR) and principal component analysis (PCA). MLR had been used widely in the early stage of biological age study; however, since several shortcomings, such as overestimation of biological age for younger people, and an underestimation of biological age for older people, have been raised [11–13], PCA algorithm has become an alternative statistical tool to compute biological age [6, 9, 10, 14]. In recent years, a new and somewhat complicated algorithm, developed by Klemera and Doubal, [15] has been used by some researchers [16, 17].

Several studies have been conducted to use biological age as an index for work ability [16], frailty [18], or physical fitness [19]. Levine and Crimmins has shown that biological age could predict 10 year mortality more accurately than other measures, such as Allostatic Load and Framingham Risk Score using 9,942 subjects [20], and shown that differences in biological age relative to chronological age account for disparities in mortality between black and white subjects [21]. In the Mennonite community in Kansas in USA, a close relationship between biological age and mortality has been investigated using 1,009 and 568 subjects, respectively [22, 23]. Meanwhile, Brown and McDaid [24] reviewed a number of studies and identified several risk factors affecting mortality in elderly person: socioeconomic/demographic risk factors such as age, education, gender, income, marital status, occupation, race/ethnicity, religion, and behavioral risk factors such as smoking, alcohol intake, physical activity, and obesity. Based on several risk factors of socioeconomic/demographic and behavioral risk factors, a mortality prediction model has been developed using a Markov chain framework and logistic regression [25]. Zhu et al. have presented a logistic regression model to gain efficiency and effectiveness in addressing a spectrum of mortality risk assessment issues based on US insure mortality experience study using 9 risk factors including gender, smoking status, issue age, and underwriting class [26].

## Methods

### Study population

The Korean Metabolic Syndrome Mortality Study (KMSMS) is a retrospective cohort study based on private health examination from 1994 to 2004, which included 557,940 Koreans of 20–93 years old who received routine total health check-ups at 15 health examination centers in Korea. The causes of death in Korea were derived from the Korean Statistics Information Service (KOSIS) database. The database was confirmed and guaranteed by national death records from the Statistics Korea (KOSTAT), the national agency of Korea. The KOSTAT estimates that its registry includes more than 99% of the deaths in Korea; cause of death was available beginning in 1992. The KOSTAT records provide the residence registration number (RRN; a unique 13-digit number assigned to all Koreans), the cause of death (International Classification of Diseases, 10th Edition), and the date of death. In Korea, all people must register the birth and death by law. The death certificate including the cause of death was almost written by doctors. Therefore, the cause of death is very reliable. The study data were merged with Cause of Death Database by residence registration number up to the year 2011. The follow-up time for the study population was a minimum of 7 years and extended up to 17 years.

From the total of 15,732 deceased people, 13,106 cases were classified as intrinsic death by referencing a previous research [27]: the numbers of death caused by cancer and non-cancerous diseases were 7,250 and 5,856, respectively. Extrinsic causes of death, such as suicide, accident, infectious disease, pregnancy-related death, or unknown causes were excluded from the study (Additional file 1: Table S1). The research proposal was approved by the Institutional Review Board of Human Research of Yonsei University.

### Construction of PCA model for biological age

The statistical engine for computing biological age was developed using a data set including total 469,754 Koreans (277,029 men and 192,725 women), which is different from MSMS data, based on PCA algorithm. From over 60 variables measured in the general health check-up, a total of 15 variables were selected as biomarkers, including WC, systolic blood pressure (SBP), diastolic blood pressure (DBP), FEV1, gamma GTP (G-GTP), BUN, HDL, LDL, TG, fasting blood sugar (FBS), erythrocyte sedimentation rate (ESR), BMI, BFP, BMP, and albumin/globulin ratio (AGR). The biomarker selection process was based on the stepwise variable selection method by maximizing coefficient of determination, which was computed using chronological age and biological age as the dependent variable and independent variable, respectively. More detailed explanation regarding the construction of PCA models to compute biological age is shown in the Additional file 1 (Appendix in Additional file 1). By using the constructed model, biological ages of the subjects consisting of MSMS data were computed at the baseline survey. The age increment of biological age over chronological age was defined as AgeDiff = biological age – chronological age for each subject: AgeDiff was used as a covariate for logistic regression analysis and Cox proportional hazards regression analysis to assess the absolute effect of biological age on mortality.

### Statistical analysis

SAS software, Version 9.2.0 (SAS Institute Inc., Cary, NC) and R language ver. 3.0.1 (R Foundation for Statistical Computing, Vienna, Austria) were used for all statistical analyses. If there were missing values in the biomarkers, population averages of the biomarkers measured in the relevant age and gender group were used. Binary logistic regression analysis and chi-square statistical tests were used to analyze the influence of biological age on the mortality adjusted by gender and chronological age. For estimating 17 years risk of mortality affected by age increment of biological age over chronological age (i.e. AgeDiff), Cox proportional hazards regression models were constructed for total subjects and for subgroups, considering chronological age and gender. Age increments of biological age over chronological age were categorized into 3 subgroups (AgeDiff < 2, 2 ≤ AgeDiff < 5, and AgeDiff ≥ 5) to estimate the discrete effect of biological age on mortality. Kaplan-Meier analysis with log rank test was applied to analyze and compare the survival probabilities of subgroups defined by AgeDiff.

## Results

### Construction of PCA model

Two PCA models were constructed to compute biological ages of men and women, respectively, which consisted of four unrotated principal components with corresponding eigen values of ≥ 1.0. About 55% of total variance was explained by these four principal components for men, and 53% of total variance for women, respectively. More detailed results about the construction of PCA model is shown in the Additional file 1 (Appendix in Additional file 1).

### Descriptive statistics of the biomarkers

From the total 557,940 subjects, 316,848 were men (56.8%) and 241,092 were women (43.2%). Average chronological ages of men and women at the baseline survey were 43.5 years and 43.6 years, respectively, and average death ages in men and women were 64.5 years and 65.4 years, respectively (Table 1). The baseline chronological ages were significantly different between alive and deceased subjects for both men and women (two sample *t*-test, *p* < 0.001). The weight of body muscle could not be obtained from the MSMS data, so population averages and standard deviations of BMP were estimated using a different data set (refer to Appendix in Additional file 1 for more detailed information). The proportions of the data which were used for the analysis excluding missing data were between 4.6% and 99.8%; 14.4% (WC), 99.8% (SBP), 92.4% (FEV1), 94.9% (G-GTP), 98.8% (BUN), 90.4%(HDL), 98.7% (TG), 99.7% (FBS), 90.7% (LDL), 21.2% (BFP), 0% (BMP), and 94.3% (AGR) for male data, and 16.5% (WC), 99.3% (DBP), 92.1% (FEV1), 93.5% (G-GTP), 98.6% (BUN), 88.6% (HDL), 98.5% (TG), 99.7% (FBS), 4.6% (ESR), 88.9% (LDL), 99.8% (BMI), 20.2% (BFP), 0% (BMP), and 93.0% (AGR) for female data. The overall distribution of biomarkers is described in the Additional file 1: Table S2. The numbers of deceased subjects whose cause of death by cancer and non-cancerous diseases were 7,250 and 5,856, respectively (Additional file 1: Table S3). Death by senility was regarded as death by non-cancerous disease, because too few cases were observed to be analyzed separately (127 cases in men and 80 cases in women, data not shown).

**Table 1:** Baseline characteristics of the study subjects

		Men (*n* = 316,848, 56.8%)			Women (*n* = 241,092, 43.2%)		
		Alive (*n* = 307,571, 97.1%)	Deceased (*n* = 9,277, 2.9%)	Total (*n* = 316,848, 100%)	Alive (*n* = 237,263, 98.4%)	Deceased (*N* = 3,829, 1.6%)	Total (*n* = 241,092, 100%)
Biomarkers	Variables	Mean ± SD	Mean ± SD	Mean ± SD	Mean ± SD	Mean ± SD	Mean ± SD
Chronological age, years	AGE	43.1 ± 10.4	58.1 ± 11.3	43.5 ± 10.7	43.4 ± 11.8	58.7 ± 11.6	43.6 ± 12.0
Age of death, years	DAGE	-	64.5 ± 11.8	64.5 ± 11.8	-	65.4 ± 12.3	65.4 ± 12.3
Waist circumference, cm	WC	86.5 ± 8.9	85.7 ± 9.9	86.5 ± 8.9	79.1 ± 11.1	82.1 ± 10.4	79.2 ± 11.1
Systolic blood pressure, mmHg	SBP	122.4 ± 16.0	131.0 ± 20.8	122.7 ± 16.2	-	-	-
Diastolic blood pressure, mmHg	DBP	-	-	-	73.2 ± 11.5	78.7 ± 13.9	73.3 ± 11.6
Forced expiratory volume in 1 s, mL	FEV1	3105.2 ± 475.4	2581.4 ± 499.3	3089.9 ± 484.3	2244.6 ± 410.4	1820.1 ± 425.2	2238.0 ± 414.0
gamma GTP, U/L	G-GTP	42.8 ± 36.3	52.3 ± 49.1	43.0 ± 36.7	18.6 ± 16.7	27.0 ± 29.2	18.8 ± 17.0
Blood urea nitrogen, mg/dL	BUN	14.7 ± 3.4	15.4 ± 4.6	14.7 ± 3.5	12.9 ± 3.4	14.5 ± 4.6	12.9 ± 3.4
High density lipoprotein, mg/dL	HDL	48.2 ± 10.0	47.8 ± 12.5	48.2 ± 10.1	55.0 ± 12.0	51.9 ± 13.3	55.0 ± 12.0
Low density lipoprotein, mg/dL	LDL	115.6 ± 32.3	116.7 ± 36.0	115.6 ± 32.5	113.4 ± 33.3	126.2 ± 39.1	113.6 ± 33.5
Triglyceride, mg/dL	TG	155 ± 96.5	153.6 ± 101.2	154.9 ± 96.6	109.8 ± 68.9	142.8 ± 89.9	110.4 ± 69.4
Fasting blood sugar, mg/dL	FBS	94.5 ± 21.4	105.9 ± 34.6	94.8 ± 22.0	90.5 ± 17.7	101.7 ± 31.3	90.7 ± 18.0
Erythrocyte sedimentation rate, mm/h	ESR	-	-	-	14.0 ± 11.4	25.6 ± 19.9	14.2 ± 11.7
Body mass index, kg/cm^2^	BMI	-	-	-	22.7 ± 3.1	24.0 ± 3.3	22.8 ± 3.2
Body fat percentage, %	BFP	29.2 ± 7.2	30.6 ± 7.9	29.2 ± 7.2	36.4 ± 6.9	38.3 ± 7.1	36.4 ± 6.9
Body muscle percentage, %	BMP	71.4 ± 7.5	71.4 ± 7.5	71.4 ± 7.5	64.6 ± 8.0	64.6 ± 8.0	64.6 ± 8.0
Albumin/globulin ratio	AGR	1.6 ± 0.2	1.6 ± 0.2	1.6 ± 0.2	1.6 ± 0.2	1.5 ± 0.2	1.6 ± 0.2

*DAGE* age of death

*BFP* weight of body fat/total weight * 100 (%)

*BMP* weight of body muscle/total weight * 100 (%)

*AGR* albumin/Globulin, *SD* standard deviation

### Distribution of age increment of biological age over chronological age

The biological ages of the subjects at the baseline survey and AgeDiff were computed as described in the Methods. The average biological age and chronological age were nearly the same in the alive subjects, however, biological age was larger than chronological age in the deceased subjects: in the deceased subjects, average AgeDiffs were 0.39 years both in men and women, which were significantly larger than the average AgeDiffs for the alive subjects, which were nearly zero (*p* value < 0.001, Table 2). AgeDiff was the largest in the subjects whose baseline chronological age was within 50–59 years (AgeDiff = 0.51 ± 1.93), and smallest in the people whose baseline chronological age was within 20–39 years (AgeDiff = 0.19 ± 2.14). In the oldest subgroup (baseline chronological age ≥ 60 years), AgeDiff was smaller than the other subgroups except 20–39 years subgroup (AgeDiff = 0.34 ± 1.66). However, the distribution of AgeDiff was different between men and women: in women, AgeDiff was smallest in the 40–49 years subgroup (AgeDiff = 0.31 ± 2.17). Meanwhile, when the cause of death was considered, AgeDiffs were computed to be larger in the subjects whose cause of death was non-cancerous disease, rather than cancer, regardless of chronological age subgroup or gender. When AgeDiff was categorized into 2 subgroups, 261,525 subjects were AgeDiff ≤ 0 (49.3%) and 268,495 were AgeDiff > 0 (50.7%) in alive subjects. In the deceased subjects, the proportions changed to 41.9% and 58.1%, respectively. When AgeDiff was categorized into 3 subgroups of AgeDiff < 2, 2 ≤ AgeDiff < 5, and AgeDiff ≥ 5, the proportions became 88.5%, 10.8%, and 0.7%, respectively in alive subjects. Meanwhile, in the deceased subjects, the proportions changed to 83.5%, 14.9%, and 1.6%, respectively: the ratios of deceased subjects relative to alive subjects significantly increased from 2.2% to 5.1% as AgeDiff became larger, whereas, ratios of alive subjects decreased from 97.8% to 94.9% as AgeDiff became larger (linear trend test, *p* value < 0.0001, Table 3). Similar patterns were observed when AgeDiff was categorized into 2 subgroups (AgeDiff ≤ 0 and AgeDiff > 0, data not shown). The trends of proportions separated by the cause of death and gender were presented in the Additional file 1: Figure S1.

**Table 2:** Average and standard deviation of AgeDiff according to gender and chronological age subgroup at the baseline survey

		Men			Women			Total		
Subject	Chronological age subgroups	*N*	Mean ± SD	*p* value	*N*	Mean ± SD	*p* value	*N*	Mean ± SD	*p* value
Alive	20-39	125,149	0.00 ± 1.65	-	97,771	0.00 ± 1.87	-	222,920	0.00 ± 1.75	-
	40-49	104,011	−0.01 ± 1.77	-	65,572	0.00 ± 2.00	-	169,583	−0.01 ± 1.86	-
	50-59	53,618	−0.03 ± 1.73	-	49,006	−0.01 ± 1.95	-	102,624	−0.02 ± 1.84	-
	≥60	24,793	−0.06 ± 1.57	-	24,914	−0.03 ± 1.72	-	49,707	−0.05 ± 1.65	-
	Total	307,571	−0.01 ± 1.70	-	237,263	−0.01 ± 1.91	-	544,834	−0.01 ± 1.79	-
Deceased by cancer	20-39	296	−0.03 ± 1.84	0.722^(1)^	210	0.30 ± 2.37	0.021^(1)^	506	0.1 ± 2.08	0.183^(1)^
	40-49	948	0.20 ± 1.77	<0.001^(1)^	324	0.11 ± 2.02	0.319^(1)^	1,272	0.18 ± 1.83	<0.001^(1)^
	50-59	1,700	0.34 ± 1.73	<0.001^(1)^	652	0.19 ± 1.93	0.008^(1)^	2,352	0.3 ± 1.79	<0.001^(1)^
	≥60	2,272	0.15 ± 1.48	<0.001^(1)^	848	0.10 ± 1.75	0.039^(1)^	3,120	0.14 ± 1.56	<0.001^(1)^
	Total	5,216	0.21 ± 1.64	<0.001^(1)^	2,034	0.15 ± 1.92	<0.001^(1)^	7,250	0.20 ± 1.72	<0.001^(1)^
Deceased by non-cancerous disease	20-39	253	0.16 ± 2.09	0.258^(2)^	83	0.76 ± 2.60	0.024^(2)^	336	0.31 ± 2.24	0.024^(2)^
	40-49	655	0.74 ± 1.96	<0.001^(2)^	142	0.77 ± 2.41	<0.001^(2)^	797	0.75 ± 2.05	<0.001^(2)^
	50-59	990	0.83 ± 1.99	<0.001^(2)^	423	0.89 ± 2.38	<0.001^(2)^	1,413	0.85 ± 2.11	<0.001^(2)^
	≥60	2,163	0.52 ± 1.67	<0.001^(2)^	1,147	0.57 ± 1.87	<0.001^(2)^	3,310	0.53 ± 1.74	<0.001^(2)^
	Total	4,061	0.61 ± 1.83	<0.001^(2)^	1,795	0.67 ± 2.08	<0.001^(2)^	5,856	0.63 ± 1.91	<0.001^(2)^
Deceased by cancer and non-cancerous disease	20-39	549	0.06 ± 1.96	0.423^(3)^	293	0.43 ± 2.44	<0.001^(3)^	842	0.19 ± 2.14	0.020^(3)^
	40-49	1,603	0.42 ± 1.87	<0.001^(3)^	466	0.31 ± 2.17	0.001^(3)^	2,069	0.40 ± 1.94	<0.001^(3)^
	50-59	2,690	0.52 ± 1.84	<0.001^(3)^	1,075	0.47 ± 2.14	<0.001^(3)^	3,765	0.51 ± 1.93	<0.001^(3)^
	≥60	4,435	0.33 ± 1.58	<0.001^(3)^	1,995	0.37 ± 1.83	<0.001^(3)^	6,430	0.34 ± 1.66	<0.001^(3)^
	Total	9,277	0.39 ± 1.74	<0.001^(3)^	3,829	0.39 ± 2.02	<0.001^(3)^	13,106	0.39 ± 1.82	<0.001^(3)^

*AgeDiff* biological age – chronological age

*p value* ^(1)^ computed using two sample *t* test, between alive subjects and deceased subjects by cancer

*p value* ^(2)^ computed using two sample *t* test, between alive subjects and deceased subjects by non-cancerous disease

*p value* ^(3)^ computed using two sample *t* test, between alive subjects and deceased subjects by cancer and non-cancerous disease

**Table 3:** Distribution of deceased or alive subjects according to gender and subgroups of AgeDiff

Subject	Subgroup	Alive, N (%)	Deceased, N (%)	*p* value
Men	AgeDiff < 2	277,136 (97.25)	7,851 (2.75)	<0.0001
	2 ≤ AgeDiff < 5	29,008 (95.69)	1,305 (4.31)	
	AgeDiff ≥ 5	1,427 (92.18)	121 (7.82)	
	AgeDiff ≤ 0	144,255 (97.50)	3,698 (2.50)	<0.0001
	AgeDiff > 0	154,204 (96.66)	5,333 (3.34)	
Women	AgeDiff < 2	205,097 (98.51)	3,101 (1.49)	<0.0001
	2 ≤ AgeDiff < 5	29,812 (97.89)	644 (2.11)	
	AgeDiff ≥ 5	2,354 (96.55)	84 (3.45)	
	AgeDiff ≤ 0	117,270 (98.61)	1,653 (1.39)	<0.0001
	AgeDiff > 0	114,291 (98.20)	2,091 (1.80)	
Total	AgeDiff < 2	482,233 (97.78)	10,952 (2.22)	<0.0001
	2 ≤ AgeDiff < 5	58,820 (96.79)	1,949 (3.21)	
	AgeDiff ≥ 5	3,781 (94.86)	205 (5.14)	
	AgeDiff ≤ 0	261,525 (97.99)	5,351 (2.01)	<0.0001
	AgeDiff > 0	268,495 (97.31)	7,424 (2.69)	

*AgeDiff* biological age – chronological age

*p value* computed using linear trend test

### Influence of AgeDiff on mortality and survival analysis

Significant influence of AgeDiff on mortality was found by binary logistic regression analysis. In men, if biological age was computed to be larger than chronological age by 1 year (i.e. AgeDiff increased by 1), the mortality was increased by 17.3% (OR = 1.173, 95% CIs = 1.158-1.189, *p* value < 0.001, Additional file 1: Table S4). In women, the mortality was increased by 13.0% (OR = 1.130, 95% CIs = 1.110 - 1.150, *p* value < 0.001, Additional file 1: Table S4). The effect of AgeDiff on mortality was more evident in deceased subjects whose cause of death was non-cancerous disease, compared to cancer: OR = 1.263 for non-cancerous disease and OR = 1.083 for cancer.

**Fig. 1:**
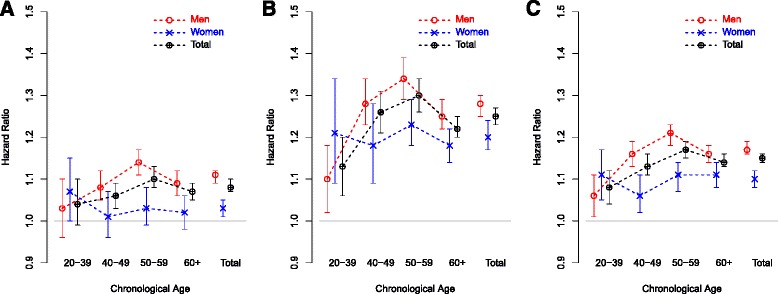
Distribution of hazard ratios according to subgroups of baseline chronological age, gender, and cause of death. Means and 95% confidence intervals are plotted. **a** Distribution of hazard ratios when the cause of death was cancer. **b** Distribution of hazard ratios when the cause of death was non-cancerous disease. **c** Distribution of hazard ratios when the cause of death was cancer and non-cancerous disease

Table 2 shows that averages of AgeDiff were different according to baseline chronological age subgroups. For this reason, Cox proportional hazards regression models were fitted separately for each baseline chronological age subgroup using AgeDiff as the continuous covariate (Table 4, Additional file 1: Tables S5, and S6). The hazard ratio in the people whose baseline chronological age was within 50–59 years was increased by 17% in per unit time as AgeDiff increased by 1 year, regardless of the event type: the hazard ratio was larger when death occurred by non-cancerous disease, compared to cancer (HR = 1.30 vs. HR = 1.10, adjusted by baseline chronological age and gender). The hazard ratio in the oldest chronological age subgroup (baseline chronological age ≥ 60 years) was slightly less than in the people whose baseline chronological age was within 50–59 years (HR = 1.14 vs. HR = 1.17). Except in the 20–39 years subgroup and event of death by cancer, all hazard ratios were statistically significant (*p* values < 0.001, Table 4). Figure 1 shows the distribution of hazard ratios for each baseline chronological age subgroup separated by gender and cause of death (means and 95% confidence intervals). In men, hazard ratios gradually increased as baseline chronological age increased from 20 years to 59 years, and decreased slightly in the oldest subjects (baseline chronological age ≥ 60). This increasing tendency was more distinct when the cause of death was non-cancerous disease, rather than cancer. Hazard ratio was largest in the subjects whose baseline chronological age was within 50–59 years (HR = 1.34, 95% CIs = 1.29 - 1.39), and smallest in the 20–39 years subgroup (HR = 1.10, 95% CIs = 1.02 - 1.18) (Additional file 1: Table S5). In women, however, no distinct pattern of increase or decrease in hazard ratio was observed across all baseline chronological age subgroups (Additional file 1: Table S6).

**Table 4:** Hazard ratios for total subjects (men and women) according to baseline chronological age subgroups and cause of death

	Death by cancer	Death by non-cancerous disease	Death by cancer and non-cancerous disease
Chronological age subgroups	HR (95% CIs)	HR (95% CIs)	HR (95% CIs)
20-39	1.04 (0.99-1.10)	1.13 (1.06-1.20)	1.08 (1.04-1.12)
40-49	1.06 (1.03-1.09)	1.26 (1.21-1.31)	1.13 (1.11-1.16)
50-59	1.10 (1.08-1.13)	1.30 (1.26-1.34)	1.17 (1.15-1.19)
≥60	1.07 (1.05-1.09)	1.22 (1.20-1.25)	1.14 (1.13-1.16)
Total	1.08 (1.07-1.10)	1.25 (1.23-1.27)	1.15 (1.14-1.16)

All results were computed using Cox proportional hazards regression analysis adjusted by baseline chronological age and gender

*HR* hazard ratio, *CIs* confidence intervals

*p* values < 0.001 for all baseline chronological age subgroups and event types except in the 20–39 years subgroup when the cause of death was cancer

When AgeDiff was categorized into 3 subgroups, such as AgeDiff < 2, 2 ≤ AgeDiff < 5, and AgeDiff ≥ 5, hazard ratios became larger as AgeDiff increased across all the chronological age subgroups and gender. For example, the estimated hazard ratio in the subgroup 2 ≤ AgeDiff < 5 was increased by 77% compared to the base subgroup (AgeDiff < 2) in men whose baseline chronological age was within 50–59 years (HR = 1.77, 95% CIs = 1.60 - 1.96, Additional file 1: Table S7). The largest hazard ratio was observed in men in the subgroup AgeDiff ≥ 5, whose baseline chronological age was within 50–59 years (HR = 4.79, 95% CIs = 3.66 - 6.27, Additional file 1: Table S7). Similar results were found even if the cause of death was separated by cancer or non-cancerous disease; however, the increment pattern of hazard ratios was more obvious when the cause of death was non-cancerous disease, rather than cancer (data not shown).

**Fig. 2:**
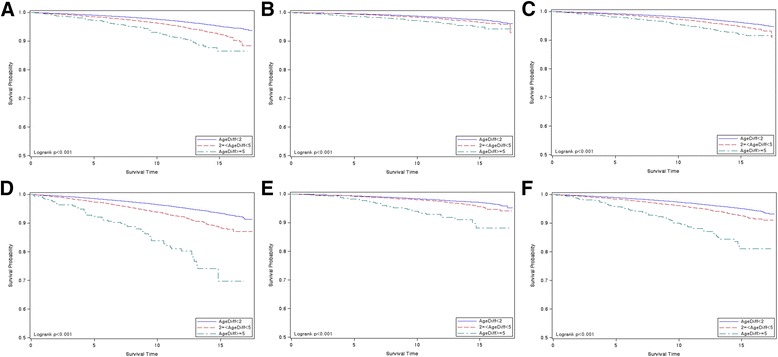
Kaplan-Meier survival plots when the cause of death included both cancer and non-cancerous disease. Blue (1), red (2), and green (3) curves are for the subjects in the AgeDiff < 2, 2 ≤ AgeDiff < 5, and AgeDiff ≥ 5 subgroups, respectively: AgeDiff = biological age - chronological age; STIME = survival time (years); Log rank test *p* value < 0.001 for all cases; **a** survival plots for men; **b** survival plots for women; **c** survival plots for men and women; **d** survival plots for men whose baseline chronological age was within 50–59 years; **e** survival plots for women whose baseline chronological age was within 50–59 years; **f** survival plots for men and women whose baseline chronological age was within 50–59 years

Kaplan-Meier analysis was performed, and survival curves were plotted to compare survival probabilities of 3 subgroups defined by AgeDiff by considering gender, event type, or baseline chronological age subgroup (Fig. 2, Additional file 1: Figures S2, S3, S4, and S5). The survival probabilities were significantly different among the 3 subgroups: in the base subgroup (AgeDiff < 2), 95.2% men survived over 15 years, whereas 92.4% and 86.5% men in the subgroup of 2 ≤ AgeDiff < 5 and AgeDiff ≥ 5 survived over 15 years, respectively (log rank test, *p* value < 0.001, Fig. 2). In women, the difference of survival probabilities was smaller than men, but still significant (log rank test, *p* value < 0.001, Fig. 2). The difference of survival probabilities among the 3 subgroups of AgeDiff was more obvious when subjects were within 50–59 years chronological age subgroup: in the base subgroup (AgeDiff < 2), 93.4% men survived over 15 years, whereas only 69.7% men in the subgroup of AgeDiff ≥ 5 survived. In women whose baseline chronological age was within 50–59 years, survival probabilities at 15 years dropped from 97.1% to 88.2% (log rank test, *p* value < 0.001, Fig. 2). Even if the cause of death was separated, survival probabilities among the 3 subgroups of AgeDiff remained significantly different, except in the case that women and the cause of death was cancer (log rank test, *p* value = 0.234, Additional file 1: Figure S2). The difference of survival probabilities was obvious across all baseline chronological age subgroups (20–39, 40–49, 50–59, and ≥ 60 years) and gender when the event was death by non-cancerous disease (log rank test, *p* value < 0.002, Additional file 1: Figure S5).

## Discussion

In the current study, we suggested that biological age could be used as a useful index to predict a person’s risk of death in the future. 15 variables were selected as biomarkers to compute biological age, as was described in the Methods section. Subjects’ biological ages were computed, using a PCA-based statistical algorithm. For the study purpose, cohort data for a total of 557,940 Koreans of 20–93 years old from MSMS was used (maximum follow-up time was 17 years). Some similar studies have been conducted [17, 20–23, 28], in which several hundreds to near ten thousand subjects have been used to compute biological ages by applying multiple linear regression, principal component analysis, or the algorithm suggested by Klemera and Doubal [15]. Then, using the same data, Cox proportional hazards regression models were constructed to estimate the effect of biological age on mortality in the future. The data size and follow-up time used in our study surpasses these previous studies. In addition, of particular importance, we have successfully validated biological age as a useful index for mortality in the future using an independently generated data set, which is different from the data set used to construct a model for computing biological age.

The finding AgeDiff was significantly greater in the deceased subjects implies that biological age might be functioning as a latent marker for mortality. Meanwhile, the increasing pattern of AgeDiff slightly declined in the oldest subgroup (baseline chronological age ≥ 60 years), which implies that the influence of biological age on the mortality diminished as people became over 60 years old. Otherwise, the biomarkers used to compute biological age did not have sufficient impact on the mortality in the oldest subjects analyzed. In the current study, the 15 biomarkers used to compute biological age have 2 types of relationship with chronological age: 11 biomarkers, including WC, SBP, DBP, G-GTP, BUN, LDL, TG, FBS, ESR, BMI, and BFP, showed a positive correlation with chronological age, and 4 biomarkers, such as FEV1, HDL, BMP, and AGR, showed a negative correlation with chronological age. A positive correlation means that a larger value is observed in the biomarker as age increases. A negative correlation means the converse. As the correlation was an overall estimation over entire chronological age ranges, age-specific correlation within some specified age range, such as chronological age ≥ 60 could be different.

In the MSMS data, 4 biomarkers, G-GTP, HDL, TG, and FBS, showed an opposite trend in deceased men: smaller values of G-GTP, TG, and FBS, and larger value of HDL were observed in the deceased subjects whose baseline chronological age ≥ 60 compared to the people of 50–59 years subgroup, which might act as diminishing factors for computing biological age. In women, smaller values of G-GTP, ESR, and BMI were observed in deceased people whose baseline chronological age ≥ 60 compared to the people of 50–59 years subgroup (data not shown). As such, it is conjectured that if different sets of biomarkers were used to compute biological age, monotonically increasing pattern of AgeDiff could be possible as the baseline chronological age becomes larger; in any case, the influence of biological age on mortality is clear in the elderly.

In addition to these results, another interesting finding is that AgeDiff was computed to be larger when the cause of death was from non-cancerous disease, compared to cancer (*p* values < 0.001, data not shown), which could be explained by the type of biomarkers used to compute biological age in this study. The majority of biomarkers were associated with metabolic syndrome, WC, SBP, DBP, HDL, TG, and FBS, or physical strength, FEV1, BFP, and BMP, but not, or little, related to cancer. Cancer-related biomarkers were only G-GTP and AGR [29, 30]. Meanwhile, the pattern of average AgeDiffs separated by baseline chronological age subgroup and/or cause of death was different between men and women. One possible explanation is that there may exist a somewhat different mechanism of senescence in men and women to influence mortality; otherwise, a biased result might be induced due to the small number of deceased female subjects, compared to men. In fact, the variances of AgeDiff in the deceased women were larger than the deceased men.

In the clinical research, chronological ages were usually categorized into several subgroups: for instance, less than 30 years, 30–60 years, and more than 60 years. In a similar way, biological age could be categorized into several subgroups; however, instead of categorizing biological age directly, the age increment, AgeDiff, was categorized into 2 or 3 subgroups, because biological age is not ‘real’, only computed as a value proxy for individual aging. As such, AgeDiff could be used to assess the absolute effect of biological age on mortality, adjusted by baseline chronological age. The ratios of deceased subjects relative to alive subjects significantly increased as AgeDiff became larger, which implies that people whose biological age was computed to be larger than their baseline chronological age were at a higher risk of mortality. The increased pattern was more apparent when the cause of death was non-cancerous disease (data not shown).

For different hazard ratios according to chronological age subgroups, gender, and cause of death, we could make some conjectures: 1) the biomarkers used to compute biological age were appropriate to assess mortality for middle to early old-aged subjects, but not for relatively younger (20–39 years) subjects, 2) there is an intrinsically different influencing mechanism of biological age on mortality between men and women, 3) current biomarkers have a relatively stronger relationship with non-cancerous disease, compared to cancer. For verification of these conjectures, a different set of biomarkers should be identified and analyzed for a mortality study jointly considering gender, chronological age, and/or cause of death.

Additional file 1: Figures S3, S4, and S5 show different decreasing patterns of survival probabilities of subjects in the different subgroups of AgeDiff, separated by baseline chronological age subgroups, gender, and cause of death. When the event was defined as death by cancer, no conspicuous difference of survival probabilities were shown in some of the baseline chronological age subgroups of men or women (*p* value > 0.2, Additional file 1: Figure S4). On the other hand, significant decreases in survival probability were observed for subgroup AgeDiff ≥ 5 when the cause of death was non-cancerous disease (*p* value < 0.002, Additional file 1: Figure S5). For instance, survival probability at 15 years was 61.7% in men whose baseline chronological age ≥ 60 when their biological age were computed as 5 years more than their chronological age. However, the survival probabilities of the subjects in AgeDiff < 2 and 2 ≤ AgeDiff < 5 subgroups were 87.6% and 76.7%, respectively, significantly larger than 61.7% (log rank test, *p* value < 0.001). These results imply that biological age could influence mortality at discrete levels, as well as in a continuous way.

In spite of the large study population and successful validation of biological age on the mortality in this study, there are limitations in this study and some improvements remain necessary. The proportions of total variance explained by the PCA models were about 50%, which is low compared to the usual case. In general, the proportion goes beyond 67% of total variance. For instance, Cho et al. reported that 66.9% of total variance had been explained by three components of which eigen values greater than 1.0 [16]. Meanwhile, Bai et al. reported that four components with eigen values ≥ 1.0 had explained 55.6% of total variance in a study based on healthy people in China [31]. In the cases when the first principal component was used alone, the proportion varied from 20.4% to 57.6% [7, 9, 10, 14, 19]. Another consideration is that different biomarkers should be identified and analyzed to assess their influence on mortality. The biomarkers used in our study were selected mainly based on the statistical correlation with the baseline chronological age. As such, it is possible that different biomarkers may explain questions that remain following our study. Finally, different algorithms should be additionally considered to compute biological age as some researchers insisted that the algorithm developed by Klemera and Doubal [15] was superior to the other algorithms in estimating biological age used here [16, 22, 28]. Despite these shortcomings, the current study remains valid as we provide strong evidence that biological age influenced mortality and could be used as a useful index to predict future risk of death by analyzing the effect of biological age on the mortality in a manifold of ways.

## Conclusions

In the current study, we suggest that biological age could be used as a useful index to predict future risk of death using a very large cohort data. Larger hazard ratios were observed in middle to old-aged people compared to younger people, which implies that biological age might be more strongly functioning as a latent marker for mortality in the senior age group. Meanwhile, larger hazard ratios were more obvious in men and when the cause of death was non-cancerous disease. However, considering the limitations and possible bias in this study, further research should be conducted in order to confirm the validity of biological age as a predictive marker for mortality.

## Additional file

Additional file 1:
**Table S1**. Inclusion criteria of cause of death for the study. **Table S2**. Overall distribution of biomarkers. **Table S3**. Number of deceased subjects according to cause of death and gender. **Table S4**. Influence of age difference (AgeDiff) on the mortality. **Table S5**. Hazard ratios for men according to chronological age subgroups and cause of death. **Table S6**. Hazard ratios for women according to chronological age subgroups and cause of death. **Table S7**. Hazard ratios for three age difference subgroups (AgeDiff) according to chronological age subgroups and gender when the event was death by cancer and non-cancerous disease. **Figure S1**. Distributions of percentage of deceased subjects according to gender, age difference (AgeDiff), and cause of death. **Figure S2**. Kaplan-Meier survival plots when the event was death by cancer or non-cancer disease. **Figure S3**. Kaplan-Meier survival plots when the event was death by cancer + non-cancer disease. **Figure S4**. Kaplan-Meier survival plots when the event was death by cancer. **Figure S5**. Kaplan-Meier survival plots when the event was death by non-cancer disease. (DOCX 678 kb)

## Abbreviations

AGR: Albumin/globulin ratio
BA: Biological age
BFP: Body fat percentage
BMI: Body mass index
BMP: Body muscle percentage
BP: Blood pressure
BUN: Blood urea nitrogen
CA: Chronological age
CIs: Confidence intervals
DBP: Diastolic blood pressure
ESR: Erythrocyte sedimentation rate
FBS: Fasting blood sugar
FEV1: Forced expiratory volume in 1 s
G-GTP: Gamma GTP
HDL: High density lipoprotein
HR: Hazard ratio
KOSIS: Korean Statistics Information Service
KOSTAT: Statistics Korea
LDL: Low density lipoprotein
MLR: Multiple linear regression
MSMS: Metabolic syndrome mortality study
OR: Odds ratio
PCA: Principal component analysis
SBP: Systolic blood pressure
TG: Triglyceride
WC: Waist circumference

## References

[1] Comfort A (1969). Test-battery to measure ageing-rate in man. Lancet.

[2] Arking R (1991). Biology of Aging: Observations and Principles.

[3] Ingram DK, Nakamura E, Smucny D, Roth GS, Lane MA (2001). Strategy for identifying biomarkers of aging in long-lived species. Exp Gerontol.

[4] Simm A, Nass N, Bartling B, Hofmann B, Silber RE, Navarrete SA (2008). Potential biomarkers of ageing. Biol Chem.

[5] Sharman A, Zhumadilov Z (2011). The scientific basis for healthy aging and antiaging process.

[6] Ueno LM, Yamashita Y, Moritani T, Nakamura E (2003). Biomarkers of aging in women and the rate of longitudinal changes. J Physiol Anthropol Appl Human Sci.

[7] Nakamura E, Miyao K (2007). A method for identifying biomarkers of aging and constructing an index of biological age in humans. J Gerontol A Biol Sci Med Sci.

[8] Bae CY, Kang YG, Kim S, Cho C, Kang HC, Yu BY, Lee SW, Cho KH, Lee DC, Lee K, Kim JS, Shin KK (2008). Development of models for predicting biological age (BA) with physical, biochemical, and hormonal parameters. Arch Gerontol Geriatr.

[9] Park JH, Cho BL, Kwon HT, Lee CM (2009). Developing a biological age assessment equation using principal component analysis and clinical biomarkers of aging in Korean men. Arch Gerontol Geriatr.

[10] Jee H, Jeon BH, Kim YH (2012). Development and application of biological age prediction models with physical fitness and physiological components in Korean adults. Gerontology.

[11] Webster IW, Logie AR (1976). A relationship between functional age and health status in female subjects. J Gerontol.

[12] Dubina TL, Dyundikova VA, Zhuk EV (1983). Biological age and its estimation. II. Assessment of biological age of albino rats by multiple regression analysis. Exp Gerontol.

[13] Hochschild R (1989). Improving the precision of biological age determinations. Part 1. A new approach to calculating biological age. Exp Gerontol.

[14] Nakamura E, Lane MA, Roth GS, Ingram DK (1998). A strategy for identifying biomarkers of aging: further evaluation of hematology and blood chemistry data from a calorie restriction study in rhesus monkeys. Exp Gerontol.

[15] Klemera P, Doubal S (2006). A new approach to the concept and computation of biological age. Mech Ageing Dev.

[16] Cho IH, Park KS, Lim CJ (2010). An empirical comparative study on biological age estimation algorithms with an application of Work Ability Index (WAI). Mech Ageing Dev.

[17] Levine ME (2013). Modeling the rate of senescence: can estimated biological age predict mortality more accurately than chronological age?. J Gerontol A Biol Sci Med Sci.

[18] Mitnitski AB, Graham JE, Mogilner AJ, Rockwood K (2002). Frailty, fitness and late-life mortality in relation to chronological and biological age. BMC Geriatr.

[19] Kimura M, Mizuta C, Yamada Y, Okayama Y, Nakamura E (2012). Constructing an index of physical fitness age for Japanese elderly based on 7-year longitudinal data: sex differences in estimated physical fitness age. Age (Dordr).

[20] Levine ME, Crimmins EM (2014). A comparison of methods for assessing mortality risk. Am J Hum Biol.

[21] Levine ME, Crimmins EM (2014). Evidence of accelerated aging among African Americans and its implications for mortality. Soc Sci Med.

[22] Uttley M, Crawford MH (1994). Efficacy of a composite biological age score to predict ten-year survival among Kansas and Nebraska Mennonites. Hum Biol.

[23] Melton PE, Zlojutro M, Kimminau K, Crawford MH (2006). Biological aging and Cox hazard analysis of mortality trends in a Mennonite community from south-central Kansas. Am J Hum Biol.

[24] Brown RL, McDaid J (2003). Factors affecting retirement mortality. N Am Actuar J.

[25] Kwon HS, Jones BL (2006). The impact of the determinants of mortality on life insurance and annuities. Insur Math Econ.

[26] Zhu Z, Li Z, Wylde D, Failor M, Hrischenko G (2015). Logistic regression for insured mortality experience studies. N Am Actuar J.

[27] Carnes BA, Holden LR, Olshansky SJ, Witten MT, Siegel JS (2006). Mortality partitions and their relevance to research on senescence. Biogerontology.

[28] Post Hospers G, Smulders YM, Maier AB, Deeg DJ, Muller M. Relation between blood pressure and mortality risk in an older population: role of chronological and biological age. J Intern Med. 2014;doi:10.1111/joim.12284. 10.1111/joim.12284 25041041

[29] Fentiman IS (2012). Gamma-glutamyl transferase: risk and prognosis of cancer. Br J Cancer.

[30] Tsutsumi M, Sakamuro D, Takada A, Zang SC, Furukawa T, Taniguchi N (1996). Detection of a unique gamma-glutamyl transpeptidase messenger RNA species closely related to the development of hepatocellular carcinoma in humans: a new candidate for early diagnosis of hepatocellular carcinoma. Hepatology.

[31] Bai X, Han L, Liu Q, Shan H, Lin H, Sun X, Chen XM (2010). Evaluation of biological aging process - a population-based study of healthy people in China. Gerontology.

