# Supplementary material for: Biological age as a useful index to predict seventeen-year survival and mortality in Koreans
Source: BMC Geriatr. 2017 Jan 5;17:7. doi: 10.1186/s12877-016-0407-y (PMC5217268; doi:10.1186/s12877-016-0407-y)
Supplement: Additional file 1: — Table S1. Inclusion criteria of cause of death for the study. Table S2. Overall distribution of biomarkers. Table S3. Number of deceased subjects according to cause of death and gender. Table S4. Influence of age difference (AgeDiff) on the mortality. Table S5. Hazard ratios for men according to chronological age subgroups and cause of death. Table S6. Hazard ratios for women according to chronological age subgroups and cause of death. Table S7. Hazard ratios for three age difference subgroups (AgeDiff) according to chronological age subgroups and gender when the event was death by cancer and non-cancerous disease. Figure S1. Distributions of percentage of deceased subjects according to gender, age difference (AgeDiff), and cause of death. Figure S2. Kaplan-Meier survival plots when the event was death by cancer or non-cancer disease. Figure S3. Kaplan-Meier survival plots when the event was death by cancer + non-cancer disease. Figure S4. Kaplan-Meier survival plots when the event was death by cancer. Figure S5. Kaplan-Meier survival plots when the event was death by non-cancer disease. (DOCX 678 kb) [file 12877_2016_407_MOESM1_ESM.docx]

**Table S1. Inclusion criteria of cause of death for the study**

| ICD code | Cause of death |
| --- | --- |
| A00-B99 | Excluded |
| C00-C97 | Cancer |
| D10-D77 | Non-Cancerous Disease |
| E00-E90 | Non-Cancerous Disease |
| F00-F99 | Non-Cancerous Disease |
| G00-G99 | Non-Cancerous Disease |
| I05-I89 | Non-Cancerous Disease |
| J00-J99 | Non-Cancerous Disease |
| K00-K93 | Non-Cancerous Disease |
| L00-L99 | Non-Cancerous Disease |
| M00-M90 | Non-Cancerous Disease |
| N00-N51 | Non-Cancerous Disease |
| O30-O92 | Excluded |
| Q20-Q64 | Excluded |
| R00-R99 | Non-Cancerous Disease |
| S00-T98 | Excluded |
| V01-Y98 | Excluded |

ICD: International Classification of Diseases

**Table S2. Overall distribution of biomarkers**

|  | Men | | | Women | | |
| --- | --- | --- | --- | --- | --- | --- |
| Variable | N (%) | Min | Max | N (%) | Min | Max |
| AGE | 316,848 (100.0) | 20 | 93 | 241,092 (100.0) | 20 | 92 |
| DAGE | 9,277 (2.9) | 26 | 98 | 3,829 (1.6) | 23 | 98 |
| WC | 45,628 (14.4) | 46 | 142 | 39,759 (16.5) | 45 | 135 |
| SBP | 316,275 (99.8) | 80 | 200 | - | - | - |
| DBP | - | - | - | 239,380 (99.3) | 50 | 185 |
| FEV1 | 292,797 (92.4) | 1000 | 7000 | 222,083 (92.1) | 1000 | 4860 |
| G-GTP | 300,706 (94.9) | 1 | 300 | 225,384 (93.5) | 1 | 300 |
| BUN | 313,045 (98.8) | 0.6 | 49.7 | 237,699 (98.6) | 2.7 | 50 |
| HDL | 286,320 (90.4) | 3 | 181 | 213,655 (88.6) | 3 | 161 |
| TG | 312,673 (98.7) | 2 | 999 | 237,376 (98.5) | 10 | 1000 |
| FBS | 316,046 (99.7) | 1.3 | 300 | 240,383 (99.7) | 3 | 300 |
| ESR | - | - | - | 11,033 (4.6) | 1 | 100 |
| LDL | 287,249 (90.7) | 0.2 | 426.8 | 214,239 (88.9) | 0.8 | 526 |
| BMI | - | - | - | 240,518 (99.8) | 15.0 | 35 |
| BFP | 67,269 (21.2) | 3.3 | 50 | 48,811 (20.2) | 3.5 | 50 |
| BMP | 0 (0.0) | - | - | 0 (0.0) | - | - |
| AGR | 298,767 (94.3) | 1 | 2.4 | 224,211 (93.0) | 1 | 2.4 |

*WC* waist circumference, *SBP* systolic blood pressure, *DBP* diastolic blood pressure, *FEV1* forced expiratory volume in 1 second, *G-GTP* gamma GTP, *BUN* blood urea nitrogen, *HDL* high density lipoprotein, *LDL* low density lipoprotein, *TG* triglyceride, *FBS* fasting blood sugar, *ESR* erythrocyte sedimentation rate, *BMI* body mass index, *BFP* body fat percentage, *BMP* body muscle percentage, *AGR* albumin/globulin ratio.

**Table S3. Number of deceased subjects according to cause of death and gender**

|  | Men | Women | Total |
| --- | --- | --- | --- |
|  | n=9,277 (70.8%) | n=3,829 (29.2%) | n=13,106 (100%) |
| Death by cancer, n (%) | 5,216 (56.2) | 2,034 (53.1) | 7,250 (55.3) |
| Death by non-cancer disease, n (%) | 4,061 (43.8) | 1,795 (46.9) | 5,856 (44.7) |

**Table S4. Influence of age difference (AgeDiff) on the mortality**

|  | Death by cancer | Death by non-cancerous disease | Death by cancer and non-cancerous disease |
| --- | --- | --- | --- |
|  | OR (95% CIs) | OR (95% CIs) | OR (95% CIs) |
| Men^(1)^ | 1.100 (1.082-1.119) | 1.279 (1.255-1.304) | 1.173 (1.158-1.189) |
| Women^(1)^ | 1.051 (1.026-1.076) | 1.237 (1.205-1.269) | 1.130 (1.110-1.150) |
| Total^(2)^ | 1.083 (1.068-1.098) | 1.263 (1.244-1.283) | 1.157 (1.145-1.170) |

*OR* odds ratio, *CIs* confidence intervals, AgeDiff = biological age – chronological age

^(1)^binary logistic regression adjusted by baseline chronological age

^(2)^binary logistic regression adjusted by baseline chronological age and gender

p values < 0.001 for all gender and event types

**Table S5. Hazard ratios for men according to chronological age subgroups and cause of death**

|  | Death by cancer |  | Death by non-cancerous disease |  | Death by cancer and non-cancerous disease |  |
| --- | --- | --- | --- | --- | --- | --- |
| chronological age subgroups | HR (95% CIs) | p value | HR (95% CIs) | p value | HR (95% CIs) | p value |
| 20-39 | 1.03 (0.96-1.10) | 0.463 | 1.10 (1.02-1.18) | 0.013 | 1.06 (1.01-1.11) | 0.027 |
| 40-49 | 1.08 (1.05-1.12) | <0.001 | 1.28 (1.23-1.34) | <0.001 | 1.16 (1.13-1.19) | <0.001 |
| 50-59 | 1.14 (1.11-1.17) | <0.001 | 1.34 (1.29-1.39) | <0.001 | 1.21 (1.18-1.23) | <0.001 |
| ≥60 | 1.09 (1.06-1.12) | <0.001 | 1.25 (1.22-1.29) | <0.001 | 1.16 (1.14-1.18) | <0.001 |
| Total | 1.11 (1.09-1.12) | <0.001 | 1.28 (1.25-1.30) | <0.001 | 1.17 (1.16-1.19) | <0.001 |

*HR* hazard ratio, *CIs* confidence intervals

All results were computed using Cox proportional hazards regression analysis adjusted by baseline chronological age

**Table S6. Hazard ratios for women according to chronological age subgroups and cause of death**

|  | Death by cancer |  | Death by non-cancerous disease |  | Death by cancer and non-cancerous disease |  |
| --- | --- | --- | --- | --- | --- | --- |
| chronological age subgroups | HR(95% CIs) | p value | HR(95% CIs) | p value | HR(95% CIs) | p value |
| 20-39 | 1.07 (1.00-1.15) | 0.059 | 1.21 (1.09-1.34) | 0.001 | 1.11 (1.05-1.17) | 0.001 |
| 40-49 | 1.01 (0.96-1.07) | 0.623 | 1.18 (1.09-1.28) | <0.001 | 1.06 (1.02-1.11) | 0.007 |
| 50-59 | 1.03 (0.99-1.08) | 0.108 | 1.23 (1.18-1.29) | <0.001 | 1.11 (1.07-1.14) | <0.001 |
| ≥60 | 1.02 (0.98-1.06) | 0.308 | 1.18 (1.14-1.22) | <0.001 | 1.11 (1.08-1.14) | <0.001 |
| Total | 1.03 (1.01-1.05) | 0.014 | 1.20 (1.17-1.24) | <0.001 | 1.10 (1.08-1.12) | <0.001 |

*HR* hazard ratio, *CIs* confidence intervals

All results were computed using Cox proportional hazards regression analysis adjusted by baseline chronological age

**Table S7. Hazard ratios for three age difference subgroups (AgeDiff) according to chronological age subgroups and gender when the event was death by cancer and non-cancerous disease**

|  | chronological age subgroups | 20-39 | 40-49 | 50-59 | ≥60 | Total |
| --- | --- | --- | --- | --- | --- | --- |
|  |  | HR(95% CIs) | HR(95% CIs) | HR(95% CIs) | HR(95% CIs) | HR(95% CIs) |
| Men^(1)^ |  |  |  |  |  |  |
|  | AgeDiff < 2 | 1 | 1 | 1 | 1 | 1 |
|  | 2 ≤ AgeDiff < 5 | 1.28(0.97-1.69) | 1.63(1.42-1.87) | 1.77(1.60-1.96) | 1.50(1.37-1.64) | 1.63(1.54-1.73) |
|  | AgeDiff ≥ 5 | 3.57(1.85-6.91) | 3.31(2.24-4.87) | 4.79(3.66-6.27) | 3.19(2.25-4.51) | 4.06(3.39-4.86) |
| Women^(1)^ |  |  |  |  |  |  |
|  | AgeDiff < 2 | 1 | 1 | 1 | 1 | 1 |
|  | 2 ≤ AgeDiff < 5 | 1.65(1.22-2.21) | 1.11(0.86-1.42) | 1.35(1.15-1.57) | 1.40(1.25-1.58) | 1.38(1.27-1.51) |
|  | AgeDiff ≥ 5 | 3.02(1.61-5.70) | 2.05(1.15-3.64) | 3.73(2.61-5.34) | 3.45(2.42-4.93) | 3.34(2.69-4.15) |
| Total^(2)^ |  |  |  |  |  |  |
|  | AgeDiff < 2 | 1 | 1 | 1 | 1 | 1 |
|  | 2 ≤ AgeDiff < 5 | 1.42(1.16-1.73) | 1.48(1.31-1.67) | 1.63(1.49-1.77) | 1.46(1.36-1.57) | 1.55(1.47-1.62) |
|  | AgeDiff ≥ 5 | 3.20(2.03-5.05) | 2.80(2.03-3.87) | 4.38(3.53-5.43) | 3.33(2.59-4.26) | 3.75(3.27-4.31) |

*HR* hazard ratio, *CIs* confidence intervals, AgeDiff = biological age – chronological age

All results were computed using Cox proportional hazards regression analysis

^(1)^adjusted by baseline chronological age

^(2)^adjusted by baseline chronological age and gender

**
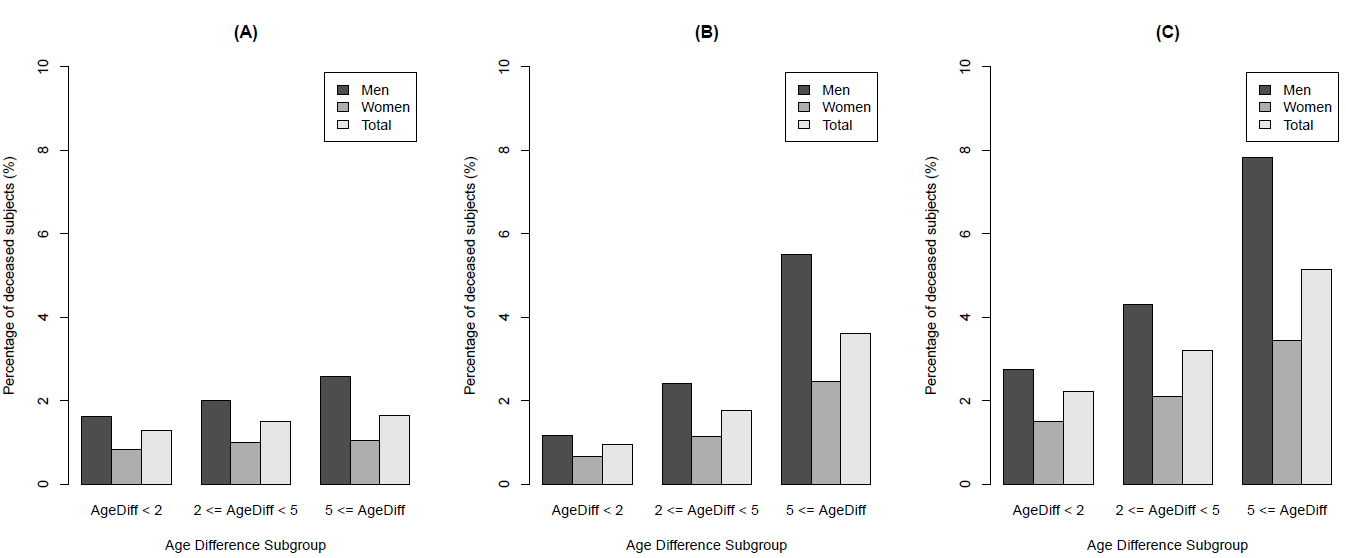
**

Figure S1. Distributions of percentage of deceased subjects according to gender, age difference (AgeDiff), and cause of death: AgeDiff = biological age – chronological age; (A) percentage of deceased subjects by cancer, (B) percentage of deceased subjects by non-cancerous disease, (C) percentage of deceased subjects by cancer and non-cancerous disease.

**
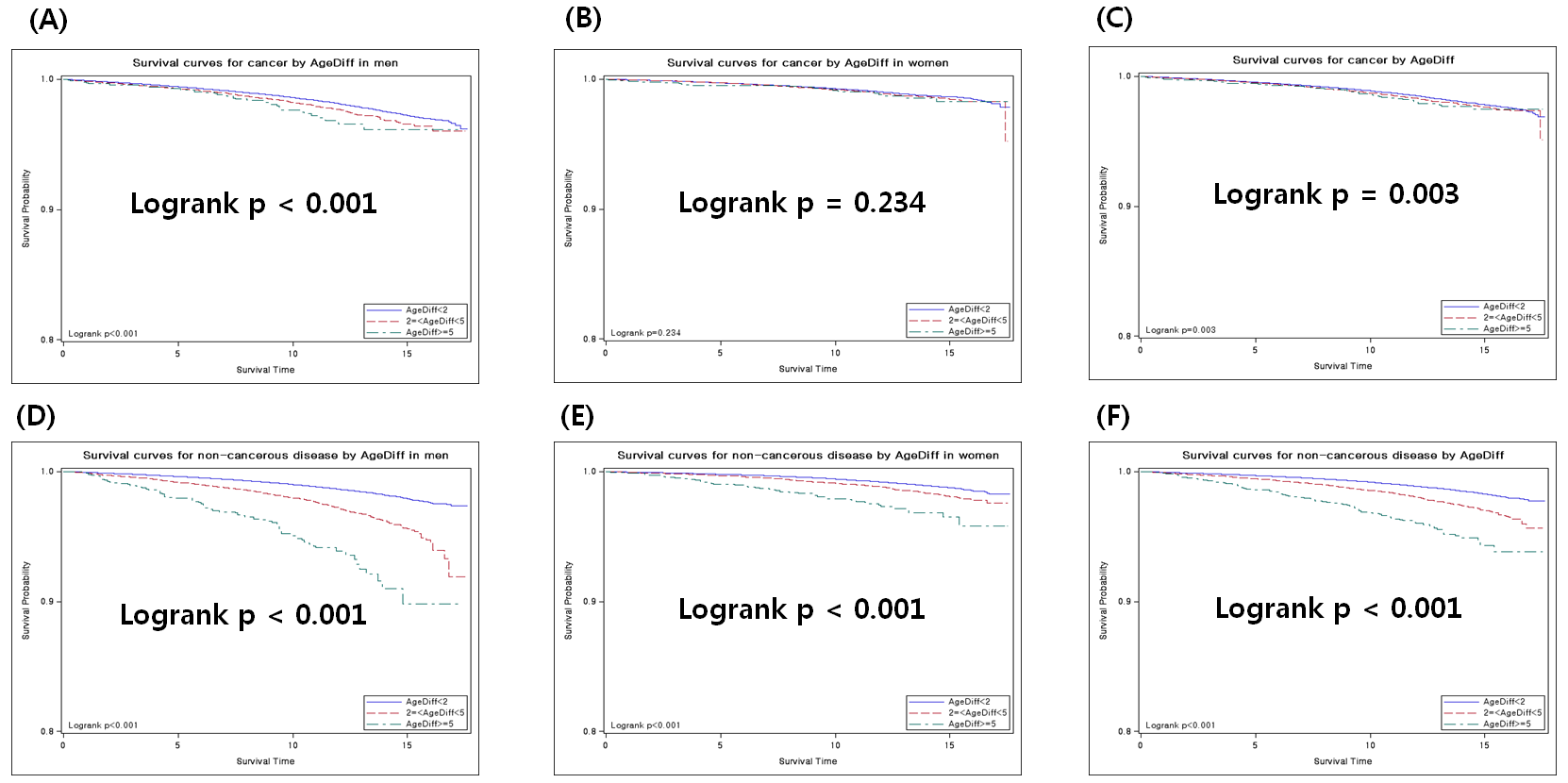
**

Figure S2. Kaplan-Meier survival plots when the event was death by cancer or non-cancer disease. Blue (1), red (2), and green (3) curves are for the subjects in AgeDiff < 2, 2 ≤ AgeDiff < 5, and AgeDiff ≥ 5 subgroups respectively: AgeDiff = biological age - chronological age; p values were computed by log rank test; STIME = survival time (years); (A) survival plots for men when the event was death by cancer; (B) survival plots for women when the event was death by cancer; (C) survival plots for men + women when the event was death by cancer; (D) survival plots for men when the event was death by non-cancer disease; (E) survival plots for women when the event was death by non-cancer disease; (F) survival plots for men + women when the event was death by non-cancer disease.

**
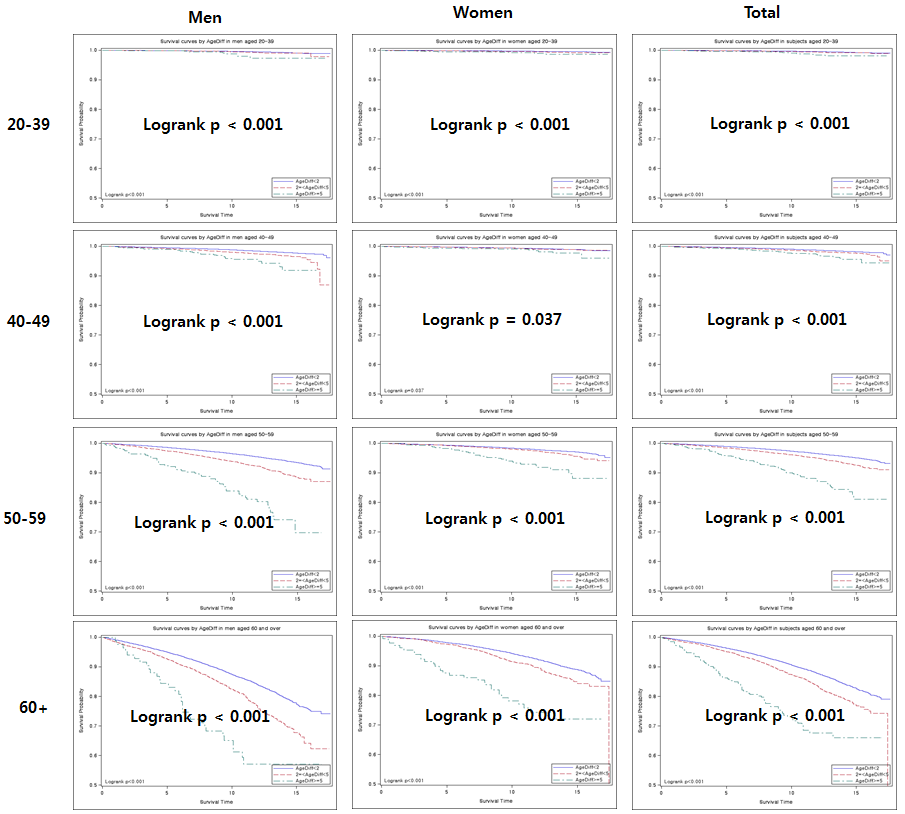
**

Figure S3. Kaplan-Meier survival plots when the event was death by cancer + non-cancer disease. Blue (1), red (2), and green (3) curves are for the subjects in AgeDiff < 2, 2 ≤ AgeDiff < 5, and AgeDiff ≥ 5 subgroups respectively: AgeDiff = biological age - chronological age; p values were computed by log rank test; STIME = survival time (years).

**
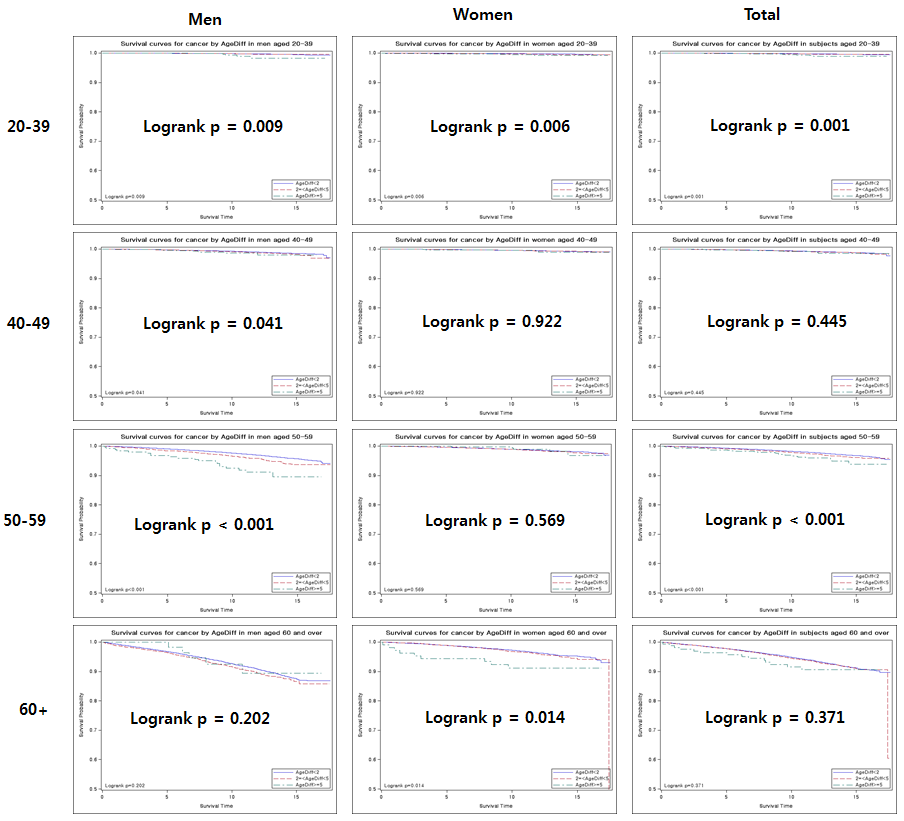
**

Figure S4. Kaplan-Meier survival plots when the event was death by cancer. Blue (1), red (2), and green (3) curves are for the subjects with age difference AgeDiff < 2, 2 ≤ AgeDiff < 5, and AgeDiff ≥ 5 respectively: AgeDiff = biological age - chronological age; p values were computed by log rank test; STIME = survival time (years).

**
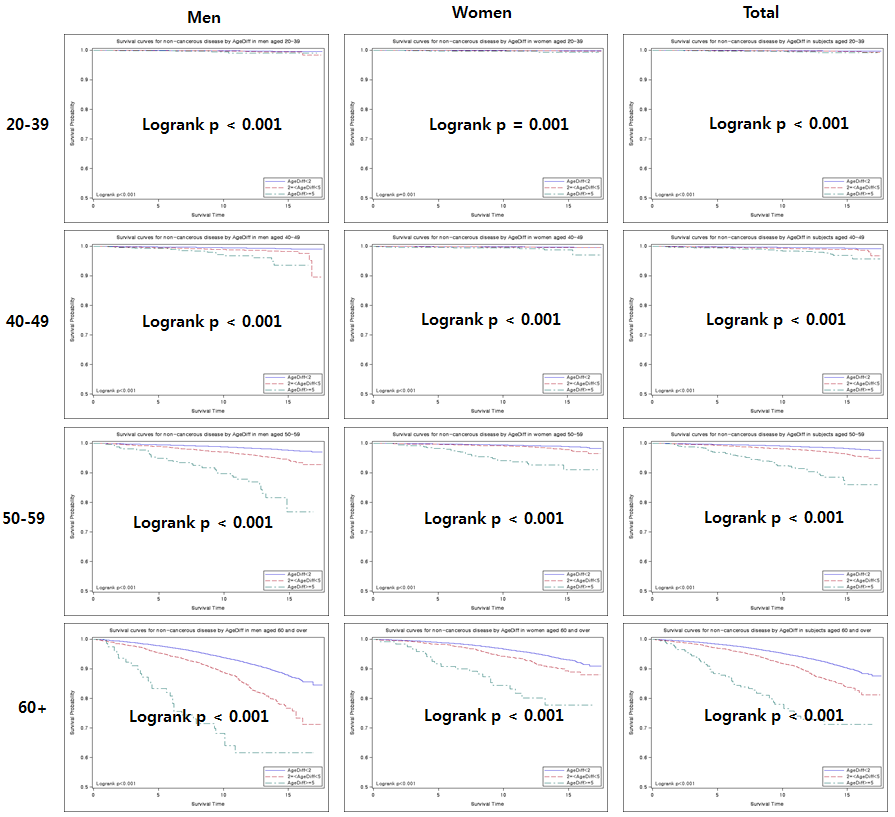
**

Figure S5. Kaplan-Meier survival plots when the event was death by non-cancer disease. Blue (1), red (2), and green (3) curves are for the subjects with age difference AgeDiff < 2, 2 ≤ AgeDiff < 5, and AgeDiff ≥ 5 respectively: AgeDiff = biological age - chronological age; p values were computed by log rank test; STIME = survival time (years).

**Appendix**

We have used a data set including 469,754 Koreans (277,029 men and 192,725 women) to construct PCA model for computing biological age, which were collected nationwide in Korea after individual consent from 2002 to 2009. This data set is different from MSMS data. From over 60 candidate variables measured in the general health check-up, total 15 variables were chosen as biomarkers to compute biological age. Figure A1 shows overall procedure to select optimized biomarkers: subjects were separated according to gender and sub-ranges of chronological age. Variables containing more than 2,000 non-missing data were considered as candidate biomarkers. Significantly correlated variables (cutoff of correlation coefficient = 0.7) were removed from the existing model to avoid multicollinearity. Then, stepwise variable selection method was performed to the remaining variables by maximizing coefficient of determination, which was computed using chronological age and biological age as dependent variable and independent variable, respectively.


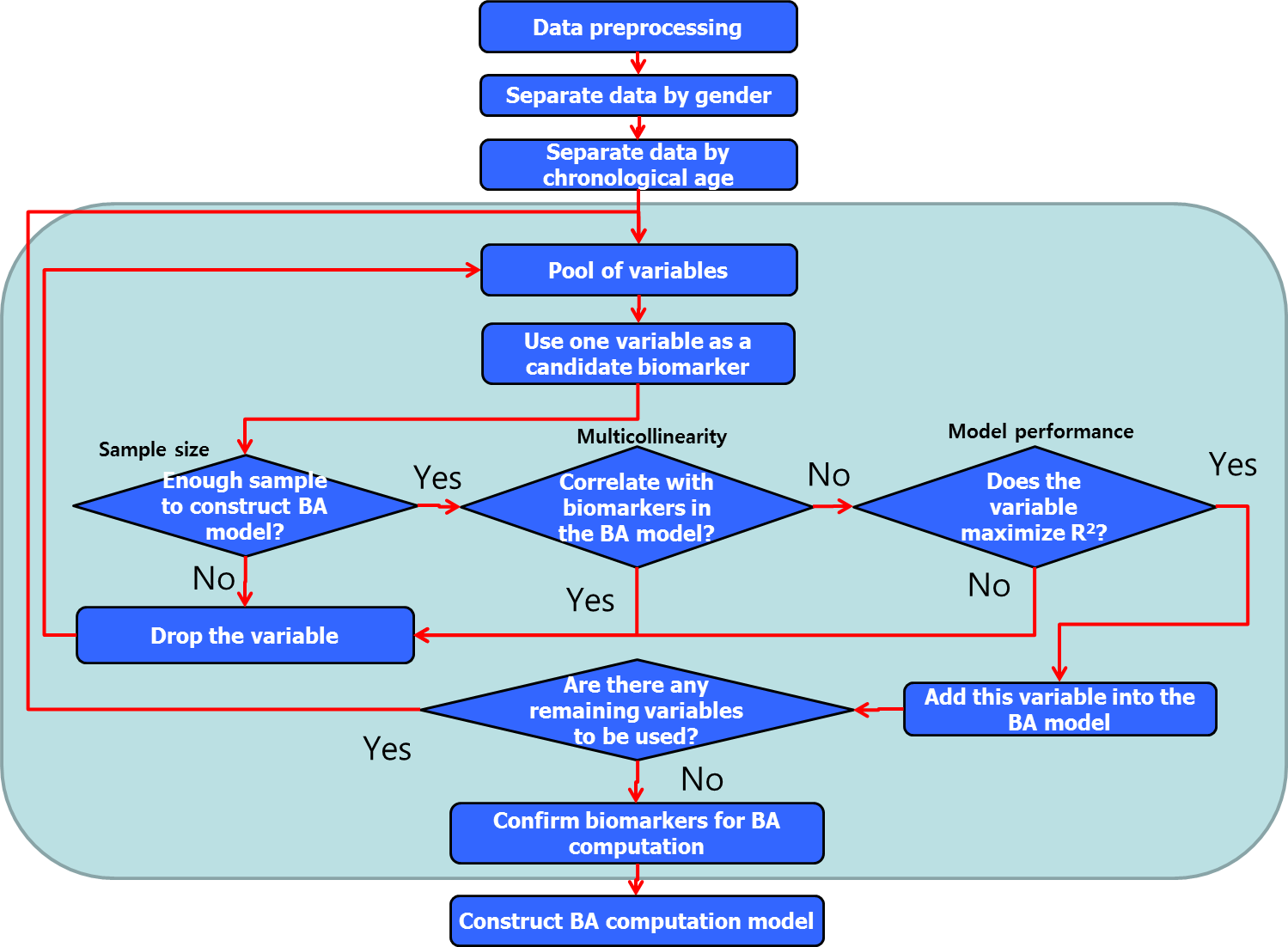


Figure A1. Overall procedure to select biomarkers for PCA model to compute biological age.

The 15 biomarkers included waist circumference (WC), systolic blood pressure (SBP), diastolic blood pressure (DBP), forced expiratory volume in 1 second (FEV1), gamma GTP (G-GTP), blood urea nitrogen (BUN), high density lipoprotein (HDL), low density lipoprotein (LDL), triglyceride (TG), fasting blood sugar (FBS), erythrocyte sedimentation rate (ESR), body mass index (BMI), body fat percentage (BFP), body muscle percentage (BMP), and albumin/globulin ratio (AGR). Biomarkers selected to compute biological age were slightly different between men and women: common biomarkers are WC, FEV1, G-GTP, BUN, HDL, LDL, TG, FBS, BFP, BMP, and AGR. SBP was unique in men and DBP, ESR, and BMI were unique in women.

Statistical engine for computing biological age was based on principal component analysis (PCA) algorithm. PCA is a statistical algorithm to reduce many variables into small number of components, which represent distinguishable characteristics of the variables in the same cluster. PCA has been used widely in computing biological age (refer to below reference). Equation 1 through equation 3 is main mathematical forms of PCA algorithm for computing biological age mainly based on reference 5. Averages and standard deviations of each variable were estimated separately according to chronological age and gender. Due to the scaless or unitless characteristic, the preBA was transformed in terms of years using T-scale idea adopted in the previous studies (reference 3 and 4). The last term, (y-$\bar{y}$)(1-$\hat{b}$), was added in the eqn.3 to correct systemic error of underestimation or overestimation of the means for biological age, which is based on the correction method suggested by Dubina et al.(reference 6). The R^2^ between preBA and chronological age were 0.343 for men and 0.420 for women, respectively. The R^2^ between biological age and chronological age were 0.705 and 0.783 for men and women, respectively.

$$preBA= \left( \sum_{i=1}^{m} \sum_{j=1}^{n} \beta_{ij}\left( \frac{x_{j}-\bar{x}_{j}}{sd\left( x_{j} \right)} \right)p_{i} \right) \text{eqn.1}$$

$$\hat{\mathrm{preBA}}=\hat{a}+\hat{b}y eqn.2$$

$$BA=preBA \times sd\left( y \right)+\bar{y}+\left( y-\bar{y} \right)\left( 1-\hat{b} \right) \text{eqn.3}$$


The final outcome is the biological age in the eqn.3. Therefore, preBA, y, sd(y), $\bar{y}$, and $\hat{b}$ should be computed previously to compute biological age. The preBA is computed using the eqn.1, which is constructed using PCA. The y, $\bar{y}$, and sd(y) indicate individual’s chronological age, average and standard deviation of chronological ages of all subjects, respectively. The $\hat{b}$ is estimated by performing a linear regression analysis where y (i.e. all subjects’ chronological age) and preBA were used as independent variable and dependent variable, respectively.

Table A1 and Table A2 show factor loadings of biomarkers used to construct PCA model for computing biological age of men and women, respectively. Table A3 and A4 show that proportion variance explained by each component of the PCA model.

[Table A1] Factor loadings of the 12 biomarkers of the PCA model for men

| Biomarker | PC1 | PC2 | PC3 | PC4 |
| --- | --- | --- | --- | --- |
| WC | 0.81 | -0.06 | -0.22 | 0.04 |
| SBP | 0.4 | 0.12 | 0.07 | 0.42 |
| FEV1 | -0.21 | -0.64 | -0.22 | -0.04 |
| G-GTP | 0.48 | -0.17 | 0.31 | 0.38 |
| BUN | 0.1 | 0.68 | 0.06 | -0.08 |
| HDL | -0.45 | 0.21 | -0.07 | 0.64 |
| TG | 0.56 | -0.34 | 0.49 | -0.1 |
| FBS | 0.36 | 0.23 | 0.47 | 0.18 |
| LDL | 0.18 | 0.21 | -0.59 | 0.17 |
| BFP | 0.81 | 0.05 | -0.25 | -0.02 |
| BMP | -0.73 | 0.09 | 0.32 | 0.01 |
| AGR | -0.19 | -0.36 | -0.07 | 0.54 |

*WC* waist circumference, *SBP* systolic blood pressure, *FEV1* forced expiratory volume in 1 second, *G-GTP* gamma GTP, *BUN* blood urea nitrogen, *HDL* high density lipoprotein, *LDL* low density lipoprotein, *TG* triglyceride, *FBS* fasting blood sugar, *BFP* body fat percentage, *BMP* body muscle percentage, *AGR* albumin/globulin ratio. Four principal components expressed as PC1, PC2, PC3, and PC4.

[Table A2] Factor loadings of the 14 biomarkers of the PCA model for women

| Biomarker | PC1 | PC2 | PC3 | PC4 |
| --- | --- | --- | --- | --- |
| WC | 0.84 | -0.29 | -0.11 | 0.08 |
| DBP | 0.49 | 0.04 | 0.19 | -0.15 |
| FEV1 | -0.43 | -0.33 | -0.38 | -0.11 |
| G-GTP | 0.37 | 0.22 | 0.19 | -0.44 |
| BUN | 0.26 | 0.34 | 0.44 | 0.47 |
| HDL | -0.02 | 0.08 | -0.04 | 0.39 |
| TG | 0.5 | 0.04 | 0.14 | -0.46 |
| FBS | 0.44 | 0.14 | 0.37 | -0.28 |
| ESR | 0.43 | 0.55 | -0.44 | -0.01 |
| LDL | 0.43 | 0.23 | 0.13 | 0.4 |
| BMI | 0.84 | -0.3 | -0.12 | 0.07 |
| BFP | 0.63 | -0.24 | -0.13 | 0.14 |
| BMP | -0.65 | 0.37 | 0.19 | -0.09 |
| AGR | -0.23 | -0.57 | 0.59 | 0.07 |

*WC* waist circumference, *DBP* diastolic blood pressure, *FEV1* forced expiratory volume in 1 second, *G-GTP* gamma GTP, *BUN* blood urea nitrogen, *HDL* high density lipoprotein, *LDL* low density lipoprotein, *TG* triglyceride, *FBS* fasting blood sugar, *ESR* erythrocyte sedimentation rate, *BMI* body mass index, *BFP* body fat percentage, *BMP* body muscle percentage, *AGR* albumin/globulin ratio. Four principal components expressed as PC1, PC2, PC3, and PC4.

[Table A3] Proportion variance explained by each component of the PCA model for men

|  | PC1 | PC2 | PC3 | PC4 |
| --- | --- | --- | --- | --- |
| Eigen Value | 3 | 1.32 | 1.19 | 1.1 |
| Proportion Variance Explained | 0.25 | 0.11 | 0.1 | 0.09 |
| Cumulative Variance Explained | 0.25 | 0.36 | 0.46 | 0.55 |

Four principal components expressed as PC1, PC2, PC3, and PC4.

[Table A4] Proportion variance explained by each component of the PCA model for women

|  | PC1 | PC2 | PC3 | PC4 |
| --- | --- | --- | --- | --- |
| Eigen Value | 3.71 | 1.35 | 1.21 | 1.09 |
| Proportion Variance Explained | 0.27 | 0.1 | 0.09 | 0.08 |
| Cumulative Variance Explained | 0.27 | 0.36 | 0.45 | 0.53 |

Four principal components expressed as PC1, PC2, PC3, and PC4.

**Reference**

1. Nakamura E, Lane MA, Roth GS, Ingram DK. A strategy for identifying biomarkers of aging: further evaluation of hematology and blood chemistry data from a calorie restriction study in rhesus monkeys. Exp Gerontol. 1998;33:421–443.

2. Ueno LM, Yamashita Y, Moritani T, Nakamura E. Biomarkers of aging in women and the rate of longitudinal changes. J Physiol Anthropol Appl Human Sci. 2003;22:37–46.

3. Park JH, Cho BL, Kwon HT, Lee CM. Developing a biological age assessment equation using principal component analysis and clinical biomarkers of aging in Korean men. Arch Gerontol Geriatr. 2009;49:7–12.

4. Jee H, Jeon BH, Kim YH, Kim HK, Choe J, Park J, Jin Y. Development and application of biological age prediction models with physical fitness and physiological components in Korean adults. Gerontology. 2012;58:344-353.

5. Yoo J, Kim Y. Biological age calculation model generation method and system thereof, biological age calculation method and system thereof. Korean Patent KR 10-1603308, 08 March 2016.

6. Dubina TL, Mints A, Zhuk EV. Biological age and its estimation. III.Introduction of a correction to the multiple regression model of biological age in cross-sectional and longitudinal studies. Exp Gerontol. 1984;19:133-143.
